# The effectiveness of using in-shoe plantar pressure assessment and monitoring in prescription therapeutic footwear to prevent plantar foot ulcer recurrence in diabetic patients: a multicenter randomized controlled trial

**DOI:** 10.1186/1757-1146-5-S1-O11

**Published:** 2012-04-10

**Authors:** Sicco A Bus, Mark LJ Arts, Roelof Waaijman, Mirjam de Haart, Tessa Busch-Westbroek, Sjef G van Baal, Frans Nollet

**Affiliations:** 1 Department of Rehabilitation, Academic Medical Centre, University of Amsterdam, Amsterdam, the Netherlands; 2 Department of Surgery, Ziekenhuisgroep Twente, location Almelo, Almelo, the Netherlands

## Background

Diabetic patients at high risk for foot ulceration are often prescribed with custom-made therapeutic footwear. However, the evidence base to support the use of this footwear for ulcer prevention is still meagre [1]. The lack of offloading efficacy may play a role in this. In-shoe plantar pressure assessment is a valuable tool for evaluating footwear and guiding modifications to optimize the footwear’s offloading properties [2]. The aim of this multicenter randomized trial was to assess the effectiveness of this approach and long-term pressure monitoring in prescription footwear to prevent plantar foot ulcer recurrence in neuropathic diabetic patients.

## Materials and methods

A total 171 neuropathic diabetic patients with a recently healed plantar foot ulcer were randomized to an intervention group that had custom-made footwear which was evaluated, optimized and monitored at 3-monthly visits using in-shoe plantar pressure analysis or a control group that had custom-made footwear which was evaluated according to current practice. Barefoot peak pressures, adherence to footwear use, and number of daily footsteps were also assessed in each patient. The primary outcome was percentage plantar foot ulcers in 18 months follow-up, which was hypothesized to be 50% lower in the intervention group than control group.

## Results

Baseline patient characteristics were not significantly different between study groups. Due to the footwear optimization approach, in-shoe peak pressures at the previous ulcer and other high pressure locations were significantly lower with ~20% in the intervention group than control group during 18 months follow-up. This is a “work-in-progress” abstract. Final data on clinical outcome will be collected early 2012 and will therefore be presented for the first time at i-FAB2012.

## Conclusion

The results of this study will provide a comprehensive view on the role of pressure offloading and other important biomechanical and behavioural factors in the prevention of foot ulceration in high-risk diabetic patients.
